# The value of bio-objects and policy discourses in Europe

**DOI:** 10.3325/cmj.2014.55.167

**Published:** 2014-04

**Authors:** Anna Lydia Svalastog

**Affiliations:** Centre for Research Ethics and Bioethics, Uppsala University, Uppsala, Sweden

What is the value of bio-objects? In a series of texts (primarily published in the *Croatian Medical Journal*) new entities, identified and/or produced by new technology, have been analytically defined as bio-objects. Bio-objects derive from innovative knowledge, they are generated through a “bio-objectification” process, and continuously negotiated in the intersection of science, politics, and society (1,2). Identification and construction of bio-objects are conducted in a variety of practices and discourses inside and outside of scientific laboratories, and configured in science, policy, and media. Information-over-loaded knowledge landscapes (KL) are in turn characterized by open-source distribution, and are both depoliticizing and (re-)politicizing the content and function of bio-objects (3,4). In this article, the focus is on the value of bio-objects, and includes an inquiry into the history of the changeable value of bio-objects as a scientific and economic project in Europe.

The organization and governance of research played an important role in the fundamental reorganization of science after World War II (WWII). Costs for research and treatment based on new technology were inevitable to achieve new knowledge, diagnostics, and treatments. Outcomes of the investments were uncertain, at the same time as priorities in the health care system became a more and more pressing issue to handle, for government as well as for individual hospitals, patient groups, and the European public. In this article, the relation between value production and society is emphasized, in particular the relative value of bio-objects, its close relation to the process of reorganizing science in post-war Europe, and the present situation of relative value generated by priority challenges.

## The scientific underpinnings of modern society

After World War II (WWII) the western world had established a new way to conduct science. The change was indeed initiated during WWII, when researchers had, for the first time, been put together to solve a given problem, the development of the atomic bomb. This project, named the Manhattan project, succeeded in its goal, ie, producing the bomb and changing the course of the war (5).

The Manhattan project represented a new mode of organization, a multidisciplinary team put together with the sole intention of solving a scientific problem that was politically defined. In addition, and for the first time science, technology, industry, and governance were working as one whole. This new structure came to be an important model for how to rearrange the relation between science, governance, and industry. In Europe, after WWII this model represented a fusion of academic work and political governance, as well as an overlap of private and public sectors (5).

## Big-(life-)science and value creation

Subsequently the focus was directed toward bio-sciences, or Life sciences (as opposed to physics, which was associated with death-science after the drop of the bombs). From the late 1970s, biotechnology was perceived as an important economic asset. It was politically framed as an enabling technology with an immense impact on industrial production processes across different sectors. Nations that were not in the forefront were said to put the wealth of their nation at peril (6). In these discourses, genetics came to represent knowledge that could generate a new era of medication, diagnostics, and therapies, in addition to a new era of agriculture, populated by new genetically modified organisms (GMO) and functional food, food as therapy and vaccine, new energy resources (energy wood), new biological solutions to pollution (bacteria that could break down oil spills and other forms of biological waste), etc. As such, these bio-objects came to represent bio-value. Though bio-objects are produced within the realm of Life sciences, they are often imbued with hopes and promises, and closely tied to imagining European identity (1).

## European identity and the Knowledge Gap Hypothesis (KGH)

The way bio-objects became part of present European identity, is explicitly linked to expected economic outcomes, both industrial as well as those related to diagnostics and medical treatments. The assumed value of bio-objects was also put in the forefront of individual EU nations as one of their main strategies for how to increase (and rescue) individual national economies. At the same time, the economic value of life sciences and new bio-objects was disputed by both the European public and the scientists, not the least the British radical science group. The disputes have shown to be complex and not reducible to questions of scientific knowledge (7-9). As such, bio-objects have an ambiguous role, tied to present ways of imagining European identity, but in a potential conflict with core European values.

A formerly accepted hypothesis was that resistance to products of new technology is a reflection of people’s lack of knowledge. The assumed discrepancy between approval by informed individuals and resistance by individuals in lack of knowledge was captured in the so-called Knowledge Gap Hypothesis (KGH). In the Biotechnology and the European Public project, the KGH did not correspond to results from new inquiries, and the KGH therefore appeared to be based on a too limited analysis (10). In addition, some topics, like controversies related to GMO and stem cell research, are not easily linked to knowledge, but clearly linked with worldviews, ethics, and religion in ways that cannot easily be solved through consensus, hence the KGH appears to carry limited explanatory value. The Biotechnology and the European Public project (9,11) (*http://ec.europa.eu/public_opinion/index_en.htm*), as well as new arenas for dialogue with the public are an example of European ambivalence and the need to find stages where controversies can be transformed into a new consensus. In democratic countries, communication to achieve clarification and consensus is an expected response to societal tensions, ambivalence, and conflict. To succeed, communication strategies need to be based on relevant understandings of why tension, resistance, ambivalence, and conflict have occurred.

## Value for whom? The relative value of bio-objects

As science became an important strategy for national and European development, research became a key area for international cooperation and new joint research centers. As one of Europe’s first joint ventures, The European Organization for Nuclear Research, physics and engineers (CERN), was established in 1954 and now contains twenty member states (*http://home.web.cern.ch/about).* In analogy with CERN, the less successful CERB, *Centre Européenne de Recherche Biologique*, was established. After the breakthroughs in DNA technology, the Human Genome Project (HUGO) was conceived in 1988, including members from 23 countries (*http://www.hugo-international.org/abt_history.php)*. In the earlier days, Life sciences referred explicitly to successes within the area of pharmaceutics, later on, due to development in different areas of molecular biology, genetics has come to the forefront, as well as chemistry (as reflected in the distributions of Nobel Prizes in the field of biology). The value of bio-objects in these arenas might be described as hyped. At the same time, due to new big-scale research and data analysis, we have seen important advances in diagnostics, pharmaceutics, and treatments, which are undisputable. Following the increasing importance of life sciences and attached industries, legal and ethical regulatory work has grown quickly, new regulatory units were established (for example the national Bio-technology Advisory Boards in Scandinavia) with a general intention to update and merge present EU regulation, as in the draft for EU Data Protection Directive. Seen from the point of view of centralized political strategies, economic priorities, and regulatory work, there seems to be a strive to fixate or essentialize the value of bio-objects.

## Human values

Research and academic life is traditionally understood as a free self-reflexive activity. Though due to the post war shift, the relation between science and politics and national economies changed. Science and academic life became a facilitator of modern society, of industry, health, and economic growth. Politics and policy turned research and academic life into means to achieve development and growth (*http://stats.oecd.org/Index.aspx?DataSetCode=MSTI_PUB*). The economy invested in knowledge and production of bio-objects represents this post war approach to science. The emphasis on science as an area of priorities was, and is, part of a situation where other areas, in particular the humanities, are put aside or even fall behind. For example, in Norway: (numbers from 2011) the humanities got 16%-17% of the students and 6%-7% of researchers/academic teachers. Natural and social sciences, represented the rest, ie, 83%-84% of the students are in natural and social sciences, and 93%-94% of Norwegian academic teachers (*http://www.regjeringen.no/nb/dep/kd/kampanjer/forskningsbarometeret/2013/mennesker/fagfordeling.html?id=728063*). In the Oslo University’s official history, the period 1975-2011 is named “Towards a new societal contract,” and it might be said that the present situation reflects the “new social contract” between society and science, which took off in the mid 1970s (12). Seen from the angle of priorities, one might ask, does the value of bio-objects imply conflicts? And is the value of bio-objects related to the lack of value in the fields of humanities? The question is made relevant by present discussions in the health sector, the same sector whose needs are used as an argument for the value of bio-objects.

The question about the value of the fields of humanities could be turned around. If we gaze at the horizon of existing bio-objects, eg, pharmaceutical products, agriculture, and GMOs, identification and distribution of genetic information, or transfer of traditional knowledge to science and industry, these activities represent fields of research that are, in order to succeed, in need of knowledge about society, history, and culture. They need the humanities either during their research process (as in epidemiological research and in pharmaceutical research transferring traditional knowledge to the laboratories), or when the product is produced and ready to be implemented in everyday life of society. From this angle, the humanities represent (should represent) a value, and also be understood as a facilitator of new bio-objects. The humanities analyze contexts, a skill that could be useful when trying to understand and solve ambiguities on bio-objects that are obviously contextually defined.

## Health, bio-economy, priorities, and the humanities

In the following, I will argue that present European health sector represents challenges concerning priorities, and that solutions need to pay more attention to the value of the humanities. Expectations about medicine and treatment in public and private health services in the last decades have changed radically due to new technology, new diagnostic tools, and new treatments related to the bio-objects. As such, health and bio-objects are tightly related. Research in the area of bio-objects and medicine is cost demanding and competence driven. Though the bio-economy is expected to represent real and expected income (in addition to increased health and well-being), the production and use of new knowledge and treatments in the area of bio-objects that the health sector is to benefit from, is increasingly expensive. Diagnostic tools and personalized medicine is evolving, though much research is still experimental or on the level of basic research on cells or mice.

The question of priorities is present and pressing in all of Europe. Sweden can be used as an example on how priorities in the health care sector generate resistance from the public as well as from medical doctors. The hospital’s priorities were meant to combine economic savings for the hospitals and democratic values (ie, be fair). Though striving to be fair, the strategy that was taken to achieve savings failed, not the least because it did not pay sufficient attention to how the value of medical treatment is relative and contextually defined; on February 17, 2013, a debate article in *Dagens Nyheter* (Today’s News) became the start of a public debate that was named “the unprofitable patient” (*Dagens Nyheter* is one of the leading national Swedish newspapers, and one of the Swedish national newspapers that were analyzed as part of the European project Biotechnology and the European Public, combining the Eurobarometer Survey on Biotechnology and national newspapers in the participating countries in their analysis) (13). The debate has centered on the existence of priority lists at certain hospitals, lists of the diseases that public hospitals are and are not to treat, surgeries that they are or are not to conduct. Priority lists were also tested, as a way to get control of economic expenses (save money) and at the same time treat patients equally. The lists represent an approach toward the patient’s needs in which diseases that are on the list are supposed to be treated by the public health care system, while diseases that are not on the list are supposed to be paid for by the patient. Doctors in the Swedish public health system opposed to this, arguing that the lists are contrary to the general principle of medicine, where the whole person has to be taken into account when deciding upon treatments (*http://www.dagensmedicin.se/nyheter/allmanmedicinarna-vagrar-skriva-prioriteringslistor-enligt-experternas-alla-regler*). What the doctors pointed out was that it was not the disease in itself but the totality of the patient’s condition and situation that one needed to evaluate. The patient and treatment cannot be understood separate from the context (ie. condition and situation). The use of technology and bio-objects in the treatment will also vary depending on condition and situation, and as such the value of technology and bio-object, in this context, will appear as context dependent.

The present debate overlaps arguments that have been used in European debate on the life sciences back in the 1950s. It concerns human value, or lack of human value, social and biological risks, and priority questions regarding research and treatment. However, the present debate in Sweden is, in comparison to former debates less general, more acute, and directly related to concrete suggestions and priorities regarding what one should treat and not treat. It stems from local situations, related to regional hospitals, their budgets, and strategies.

## Conclusion

The value of bio-objects is tentative, and the discourses of bio-objects are tightly tied to an organization of society where politics, priorities, science, and technology are fused. This has changed the former distinction between public and private spheres, and made economy and industrialization an explicit and expected outcome of research cooperation and research development. It has also implied the new relations between scientific investigation and academic freedom, medical science and clinical practice. Together with the identity of bio-object, the value of bio-objects is context-dependent. The context-bound identity and value of bio-objects makes the humanities an important facilitator for production and implementation of bio-objects. In addition, to understand present tensions and controversies regarding bio-objects and societal priorities that involve bio-objects, the humanities represent tools for analysis of context and of the complex relation between societal continuity and change.
